# Proteomic snapshot of saliva samples predicts new pathways implicated in SARS-CoV-2 pathogenesis

**DOI:** 10.1186/s12014-024-09482-9

**Published:** 2024-05-22

**Authors:** Elena Moreno, Sergio Ciordia, Santos Milhano Fátima, Daniel Jiménez, Javier Martínez-Sanz, Pilar Vizcarra, Raquel Ron, Matilde Sánchez-Conde, Rafael Bargiela, Sergio Sanchez-Carrillo, Santiago Moreno, Fernando Corrales, Manuel Ferrer, Sergio Serrano-Villar

**Affiliations:** 1 Department of Infectious Diseases, Facultad de Medicina, Hospital Universitario Ramón y Cajal, IRYCIS, Carretera de Colmenar Viejo, Km 9.100, 28034 Madrid, Spain; 2 CIBERINFEC, Instituto de Salud Carlos III, 28029 Madrid, Spain; 3 Functional Proteomics Laboratory, Centro Nacional de Biotecnología (CNB), CSIC, 28049 Madrid, Spain; 4 Centre for Environmental Biotechnology, School of Natural Sciences, Bangor University, Bangor, LL57 2UW UK; 5 Instituto de Catalisis y Petroleoquimica (ICP), CSIC, 28049 Madrid, Spain; 6 Facultad de Medicina, Universidad de Alcalá de Henares, 28801 Alcalá de Henares, Madrid Spain; 7 Present Address: Centro de Biologia Molecular Severo Ochoa (CBM), CSIC-UAM, 28049 Madrid, Spain

**Keywords:** Proteomics, SARS-CoV-2, Saliva, Pathogenesis, Functional analysis

## Abstract

**Background:**

Information on the microbiome's human pathways and active members that can affect SARS-CoV-2 susceptibility and pathogenesis in the salivary proteome is very scarce. Here, we studied a unique collection of samples harvested from April to June 2020 from unvaccinated patients.

**Methods:**

We compared 10 infected and hospitalized patients with severe (*n* = 5) and moderate (*n* = 5) coronavirus disease (COVID-19) with 10 uninfected individuals, including non-COVID-19 but susceptible individuals (*n* = 5) and non-COVID-19 and nonsusceptible healthcare workers with repeated high-risk exposures (*n* = 5).

**Results:**

By performing high-throughput proteomic profiling in saliva samples, we detected 226 unique differentially expressed (DE) human proteins between groups (q-value ≤ 0.05) out of 3376 unambiguously identified proteins (false discovery rate ≤ 1%). Major differences were observed between the non-COVID-19 and nonsusceptible groups. Bioinformatics analysis of DE proteins revealed human proteomic signatures related to inflammatory responses, central cellular processes, and antiviral activity associated with the saliva of SARS-CoV-2-infected patients (p-value ≤ 0.0004). Discriminatory biomarker signatures from human saliva include cystatins, protective molecules present in the oral cavity, calprotectins, involved in cell cycle progression, and histones, related to nucleosome functions. The expression levels of two human proteins related to protein transport in the cytoplasm, DYNC1 (p-value, 0.0021) and MAPRE1 (p-value, 0.047), correlated with angiotensin-converting enzyme 2 (ACE2) plasma activity. Finally, the proteomes of microorganisms present in the saliva samples showed 4 main microbial functional features related to ribosome functioning that were overrepresented in the infected group.

**Conclusion:**

Our study explores potential candidates involved in pathways implicated in SARS-CoV-2 susceptibility, although further studies in larger cohorts will be necessary.

**Graphical Abstract:**

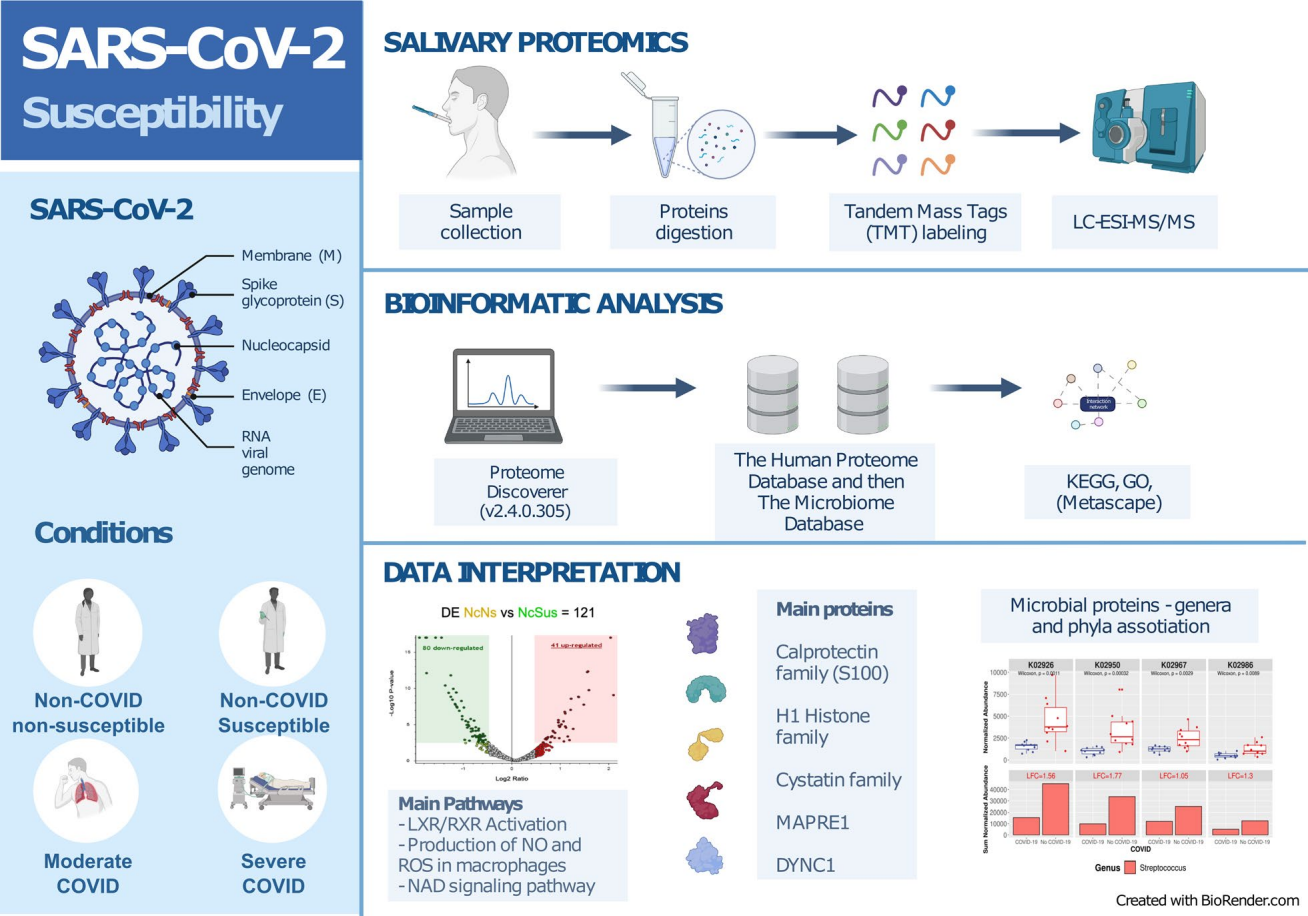

**Supplementary Information:**

The online version contains supplementary material available at 10.1186/s12014-024-09482-9.

## Introduction

Although 3 years have passed since the beginning of the pandemic caused by severe acute respiratory syndrome coronavirus 2 (SARS-CoV-2) and a myriad of publications have tried to explain the mechanisms underlying its infectious process, only a few studies have focused on human and bacterial proteomic analysis in saliva to understand SARS-CoV-2 pathogenesis. As a respiratory virus, SARS-CoV-2 primary target cells reside in the nasopharyngeal and oropharyngeal mucosa. Saliva samples are noninvasive and inexpensive to obtain, providing an ideal site to discriminate between physiological and pathological conditions [1].

The characterization of the human salivary proteome led to the identification of more than 3000 proteins, mostly of microbial origin, supporting the notion that the oral microbiota is a major contributor to the whole salivary proteome [2]. Indeed, previous metagenomic studies have detected more than 10,000 taxa in saliva [3–6]. The functional relevance of the microbiota is supported by other groups that have identified microbial peptides from patients with a specific disease and described the functions associated with those proteins in that context [7, 8]. This approach seems especially relevant in the case of a respiratory virus since the probability of direct interactions with salivary proteins is high.

The pathophysiology of SARS-CoV-2 has been associated with an abnormal immune response, and a severe response to the infection can be facilitated by several comorbidities, such as diabetes, pulmonary disease, or cardiovascular disease [9, 10]. However, the role of saliva composition in disease pathogenesis remains unclear, and research has been carried out on other types of samples, such as plasma, nasopharyngeal swabs, feces, and semen, as will be detailed in the Discussion section. To address this question, we performed an exploratory study comparing unvaccinated patients hospitalized with different degrees of severe COVID-19 disease and healthcare workers involved in COVID-19 care during the first wave in Madrid (Spain) without clinical or serologic evidence of developing infection despite repeated high-risk exposures to SARS-CoV-2. Saliva samples were collected and analyzed using a tandem mass tag (TMT)-based quantitative proteomics technique, and their proteomics profile was correlated with previously described SARS-CoV-2 susceptibility and COVID-19 clinical progression traits. Furthermore, we searched for alterations in saliva related to microbiota, which allowed us to find relevant functional bacteria associated with specific conditions. By using these complementary approaches, we show here some molecular principles describing the underlying mechanisms responsible for different susceptibilities to COVID-19, which can be relevant for the clinical management of COVID-19 patients.

## Results

### General characteristics of the study population

The study population included 20 individuals, 10 non-COVID individuals with negative polymerase chain reaction (PCR) test results and 10 hospitalized patients due to COVID-19 disease symptoms. Of the non-COVID individuals, 5 were healthcare workers considered nonsusceptible to COVID-19 (NcNs), since they remained seronegative despite repeated high-risk exposures, and 5 were healthy controls but susceptible to COVID-19 (NcSus), i.e., they became infected during the follow-up. Of the infected patients, 5 were considered moderate cases (Mcov) (i.e., they did not require respiratory support by intubation), and 5 developed severe disease (Scov) (i.e., they needed intubation). The general characteristics of the study participants are shown in Table 1, and more details are displayed in the materials and methods**.**

**Table 1:** General characteristics of the study population

Study id	Group	Age (years)	Sex	Diabetes	ObeYest	Lung disease	Cardiac disease	Outcome
24	Non-COVID nonsusceptible (NcNs)	34	Female	No	No	No	No	Healthy
31	Non-COVID nonsusceptible (NcNs)	24	Male	No	No	No	No	Healthy
16	Non-COVID nonsusceptible (NcNs)	39	Male	No	No	No	No	Healthy
22	Non-COVID nonsusceptible (NcNs)	38	Female	No	No	No	No	Healthy
59	Non-COVID nonsusceptible (NcNs)	37	Female	No	No	No	No	Healthy
65	Non-COVID susceptible (NcSus)	44	Female	No	No	No	No	Healthy
71	Non-COVID susceptible (NcSus)	38	Female	No	No	No	No	Healthy
66	Non-COVID susceptible (NcSus)	57	Female	No	No	No	No	Healthy
43	Non-COVID susceptible (NcSus)	48	Female	No	No	No	No	Healthy
45	Non-COVID susceptible (NcSus)	51	Female	No	No	No	No	Healthy
26	Moderate COVID (Mcov)	91	Female	Yes	No	No	No	Survived/no intubation
3	Moderate COVID (Mcov)	69	Male	Yes	Yes	No	No	Survived/no intubation
5	Moderate COVID (Mcov)	89	Female	No	No	No	No	Survived/no intubation
74	Moderate COVID (Mcov)	78	Male	No	No	No	Yes	Survived/no intubation
68	Moderate COVID (Mcov)	75	Female	No	No	No	No	Survived/no intubation
157	Severe COVID (Scov)	58	Male	Yes	No	No	No	Survived/intubation
162	Severe COVID (Scov)	47	Male	No	No	No	No	Survived/intubation
35	Severe COVID (Scov)	60	Female	Yes	No	Yes	No	Survived/intubation
20	Severe COVID (Scov)	92	Male	Yes	No	No	No	Deceased
177	Severe COVID (Scov)	61	Male	No	No	No	No	Survived/intubation

### Human proteins in saliva related to different SARS-CoV-2 infection sensitivities

**Fig. 1:**
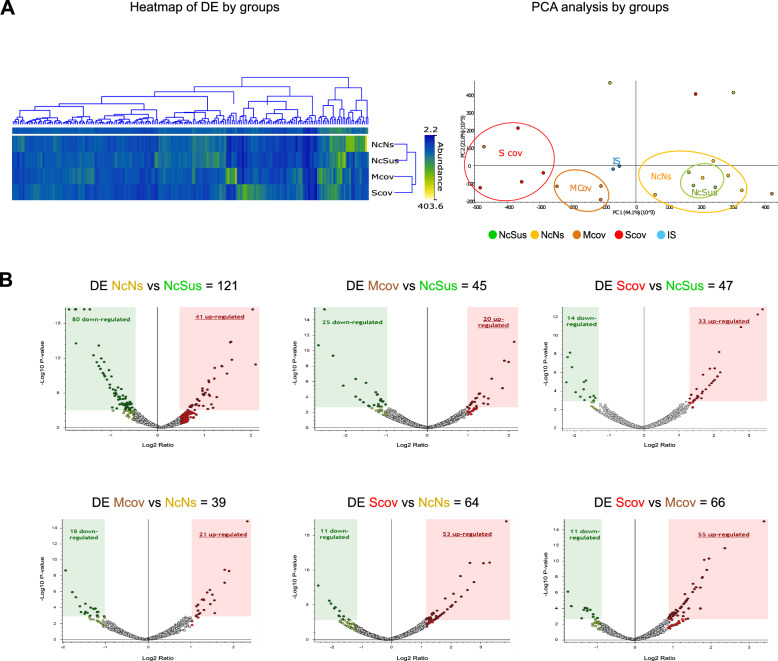
Analysis of DE proteins in the saliva of patients with COVID-19. **A** Heatmap and PCA of DE proteins (see Additional file 1: Table S1) by samples divided into 4 groups [NcSus (n = 5; green), NcNs (n = 5; yellow); Mcov (n = 5; orange); Scov (n = 5; red)] plus the IS (internal standard). A heatmap was created using normalized abundance data, and branches represent grouping by similarities. PCA for overrepresented proteins was depicted using the log_2_-transformed abundances and explained ~ 44,1% of the variance in PC1 and 21.8% in PC2. **B** Volcano plots show the proteins that are DE between the different study groups (upregulated proteins are represented in dark red and downregulated proteins in dark green). The statistical thresholds used for these analyses are detailed in the Materials and Methods section

A TMT-based proteomics approach by liquid chromatography coupled to tandem mass spectrometry (LC‒MS/MS) was applied to analyze and compare the expression levels of proteins in saliva samples of the 20 individuals conforming to the four target COVID-19 susceptibility groups (Table 1; Additional file 1: Table S1A). Combining the information obtained from the four search engines, a total of 3378 human proteins were unambiguously identified with an FDR < 1% (Additional file 1: Table S1B). Similar to other studies focused on the analysis of biological fluids by mass spectrometry during COVID-19 infection [2], SARS-CoV-2 proteins were not detected in this study. Of the 3378 proteins, a group of 2721 proteins were quantified in two TMT experiments with two Internal Standards (IS) (Additional file 1: Table S1C), and different unique proteins were found to be differentially expressed (DE) depending on the comparisons of the groups (Additional file 1: Table S1D–I). Both the representation of DE proteins on a heatmap and unsupervised principal component analysis (PCA) showed a clear separation between the non-COVID individuals (NcN and NcSus groups) and the infected patients (moderate COVID, Mcov and severe COVID, Scov groups) (Fig. 1A). Furthermore, Volcano plots show the best differences in the comparison between the NcNs and the NcSus groups (121 proteins), followed by Scov vs Mcov (66 proteins), Scov vs NcNs groups (64 proteins), Scov vs NcSus (47 proteins), Mcov vs NcSus (45 proteins), and Mcov vs NcNs (39 proteins) (Fig. 1B; Additional file 1: Table S1D–I).

### Functional analysis of DE human proteins: biological meaning of different COVID-19 disease outcomes

**Fig. 2:**
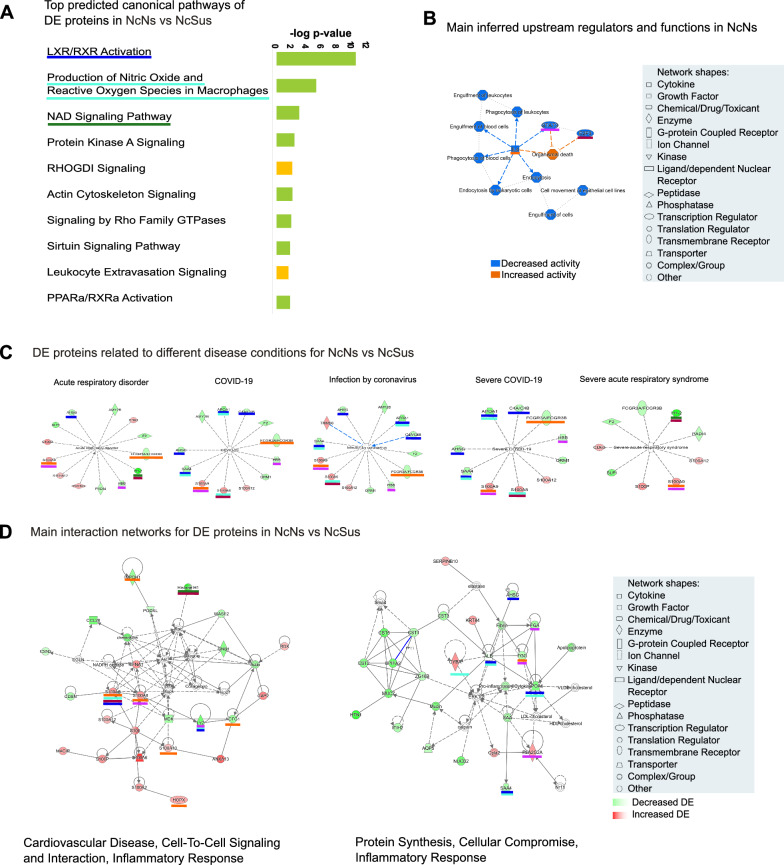
Results of the predicted functional analysis of the proteins found DE in saliva of NcNs compared NcSus patients by IPA software. **A** Top 10 canonical pathways common for the two groups by the top 10 were selected from Additional file 1: Table S2 (highlighted in yellow) by selecting the ones with the highest log_10_ ratio (observed/expected) and adjusted p-value cutoff ≤ 0.5. **B** Graphical summary with the main upstream regulators with predicted increased (orange) and decreased (blue) activity. **C** Diseases and biological functions related to DE proteins, including infectious and respiratory diseases associated with infection by SARS-CoV-2. **D** Main interaction networks and related upstream regulators/molecules predicted by IPA (white background). Upregulated molecules are depicted in red and downregulated molecules in green according to adjusted p-values of overlap calculated by IPA software and detailed in Additional file 1: Table S2

To understand the different roles of the DE human proteins found in saliva, a functional analysis was performed using Ingenuity Pathway Analysis (IPA) software (Qiagen). This analysis inferred the most significant pathways, upstream regulators, diseases, and biological functions associated with the DE proteins by comparison with the Ingenuity Knowledge database. This tool has been applied before to investigate canonical pathways of human proteomes in nasopharyngeal swabs from COVID-19 patients [11]. This analysis was performed for the DE proteins found in paired comparisons for all the groups (Additional file 1: Figure S1-5, Additional file 1: Table S2A–F). However, to understand the underlying mechanisms that could explain the different susceptibilities to COVID-19 disease, we focused on the proteins found to be DE in the non-COVID-19 nonsusceptible condition (NcNs) compared to the non-COVID-19 susceptible condition (NcSus) (Fig. 2 and Additional file 1: Table S2B). In this analysis, 10 canonical pathways showed relevant differences (− log_10_(p-value) > 1.8 and z-score value > 0, highlighted in yellow in Additional file 1: Table S2). B), such as those related to LXR/RXR activation, production of nitric oxide and reactive oxygen species (ROS) in macrophages and NAD signaling (Fig. 2A). From this same analysis, we also inferred the main upstream regulators of those DE proteins with a predicted decreased activation state in the NcNs group. These include interleukin-4 (IL-4), signal transducer and activator of transcription 3 (STAT3), and transcription factor E2F3 (E2F3), with regulatory functions related to cellular mechanisms (endocytosis and cell movement), blood cell processing (e.g., phagocytosis of leukocytes), and regulation of cell apoptosis (Fig. 2B).

Furthermore, IPA analysis of these proteins showed a pattern associated with a low susceptibility to SARS-CoV-2. Some of the proteins specifically decreasingly or increasingly expressed, which are represented by different colors, are related to COVID-19 or acute respiratory disorder in general, as shown in Fig. 2C. Of these, the expression of apolipoprotein A-I (APOA1), alpha-2-HS-glycoprotein (AHSG), hemoglobin beta chain (HBB), serum amyloid A-4 (SAA4), alpha-1-acid glycoprotein 1 (ORM1), and complement C4A (C4A) was found to be significantly reduced in the NcNs group compared to the NcSus group. Furthermore, some of these proteins were also found to be upregulated in the saliva of patients with severe and moderate COVID-19 (Scov and Mcov) when compared with susceptible and/or nonsusceptible individuals (Additional file 1: Table S1D, E, H, I and Additional file 1: Figure S1-5). Interestingly, calprotectin protein family (S-100) expression, including S100A8, S100A12, S100A9, and S100P, was found to be increased in the NcNs group compared with the NcSus group (red in Fig. 2D), in contrast to their decreased expression during COVID-19 infection conditions (Additional file 1: Table S1C-Scov or Mcov conditions abundance ratio with adj p-values ranging from 2.28 × 10^–2^ to 1.96 × 10^–5^). Furthermore, related to the predicted canonical pathways by the IPA analysis, it is worth mentioning the LXR/RXR activation pathway with APOA1, AHSG, SAA4, ORM1, S100A8, and C4A being involved and the STAT3 pathway with AHSG, HBB, and S100A9 and APOA1, SAA4, and C4A (Additional file 1: Table S2, sheet B, highlighted in yellow and Fig. 2C and D, blue underlined). These proteins are related to cholesterol transport to tissues and lipoprotein synthesis (HDL), which acts as a scavenger of viruses, immune modulator, and mediator of viral entry.

To further understand the mechanisms related to the DE proteins in the nonsusceptible group, we selected the two main interaction networks predicted in the analysis according to the IPA score and showed the main processes associated with them: they range from cell-to-cell signaling and interaction to protein synthesis, cellular compromise, and inflammatory response (Fig. 2D). Furthermore, we underlined in matching colors the main protein families in these interaction networks with the predicted canonical pathways (Fig. 2A) and/or upstream regulators (Fig. 2B). To summarize these results, the main DE proteins and predicted functions related to SARS-CoV-2 susceptibility obtained in the IPA analysis are described in Table 2.

**Table 2:** Summary of the main functions and proteins related with SARS-CoV-2 susceptibility

Proteins	Pathway or function related
S100A8, LYZ, AHSG, APOA1, and SAA4	LXR/RXR activation and production of nitric oxide and ROS in macrophages
CYBA	oxidative stress-related pathways
H1 family	NAD signaling pathway and E2F3 (upstream regulator)
S100A9, LYZ, fibrinogen chains (FGA and FGG) and PLA2G2A	STAT3 immune pathway
S100 protein family, IMPDH1, ACTG1	IL-4 (act as regulator)

**Fig. 3:**
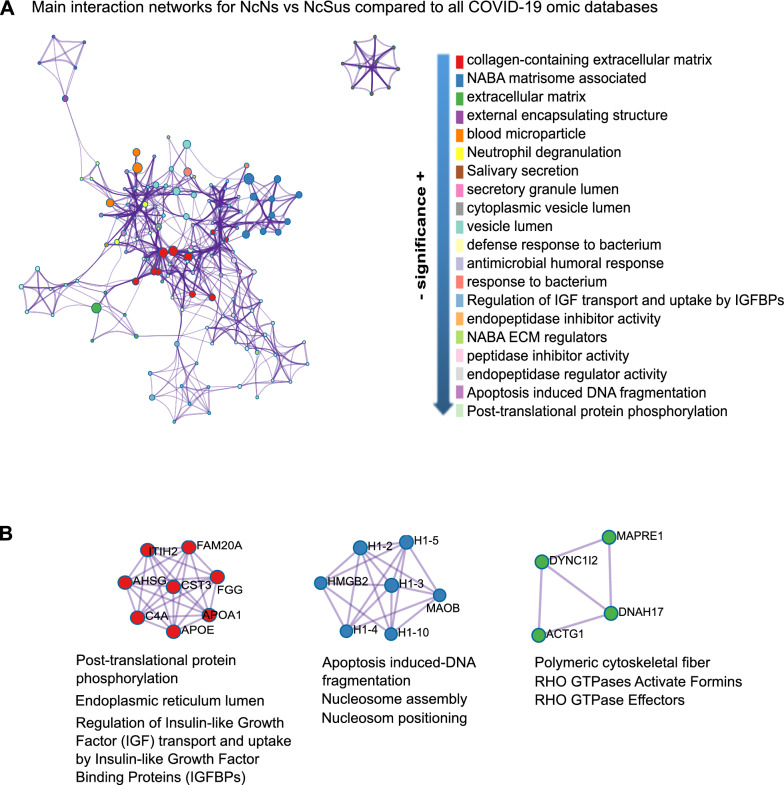
Comparison of DE proteins in the saliva of the NcNs vs NcSus groups with all omics COVID-19 databases by Metascape. **A** Main interaction network predicted by Cytoscape (left) and list ordered by more probable functions related to the DE proteins selected (right). The colors of the functions match the colors in the network. The size of the nodes represents the number of proteins associated with that function (the bigger the node, the higher the number of the selected proteins involved in that function). **B** Main subnets of the protein interactions inferred by the MCODE algorithm and their main predicted biological functions (below) according to a p-value < 0.01. Detailed information about the enrichment analysis, statistical values and specific proteins associated with the functions is further detailed in Additional file 1: Table S3 and its caption

Next, to further understand the potential role specifically in the COVID-19 context of some of the DE human proteins in nonsusceptible patients, we compared the 121 DE proteins obtained in the analysis of NcNs vs NcSus with all the -omic studies published that identified direct interactions and biological pathways related to SARS-CoV-2 by using Metascape [12]. The main discriminatory pathways were specific for microbial infections, immune response, and cellular growth and were even specific for salivary secretion (Fig. 3A). Clustering coefficients calculated using enrichment analysis and the Cytoscape and MCODE algorithms for subnet analysis identified three main clusters of proteins (Fig. 3B, Additional file 1: Table S3). The first cluster contains the above mentioned proteins related to LXR/RXR activation and cholesterol metabolism and other related proteins, such as cystatin C (CST3), interalpha-trypsin inhibitor heavy chain H2 (ITIH2), fibrinogen gamma chain (FGG), and pseudokinase FAM20A (FAM20A). Therefore, the levels of expression of these proteins follow the same tendency as the others. The second cluster is related to apoptosis-induced DNA fragmentation, nucleosome assembly and positioning and includes proteins found to be downregulated in NcNs, including several isoforms of histone H1, amine oxidase [flavin-containing] B (MAOB), and high mobility group protein B2 (HMGB2). The last cluster includes proteins involved in Rho GTPase-mediated regulation and organization of polymeric cytoskeleton rearrangement. Cytoplasmic dynein 1 (DYNC1), microtubule-associated protein RP/EB family member 1 (MAPRE1), and cytoplasmic actin, 2 (ACTG1) were downregulated in the NcNs compared to NcSus, in contrast with axonemal dynein 17 (DNAH17), whose levels were significantly higher in NcNs compared to NcSus and COVID^+^ groups. All the proteins in these subnetworks were also highlighted by IPA analysis, although some of them were not in the main networks (FAM20A, MAOB, DYNC1, MAPRE1, and DNAH17) (Fig. 3).

Furthermore, by comparison with other databases (TRRUST, Transcription Factor Targets, PaGenBase), different specific functions were found to be altered. Three relevant transcription factors, namely, SP1 (TTRUST database; also identified in the IPA analysis), which has been described as involved in viral transcription [13–16], LMO2, a regulator of T-cell translocation [17, 18], and AP1, are related to viral infection and ROS [19, 20] (Transcription Factor Targets database). Finally, the proteins were associated to some specific tissues and cell types, such as salivary gland and lung or skeletal muscle and lung cells (Figure S6; − log_10_(p-value) > 4.0).

### Microbiome composition and role in SARS-CoV-2 infection susceptibility

**Fig. 4:**
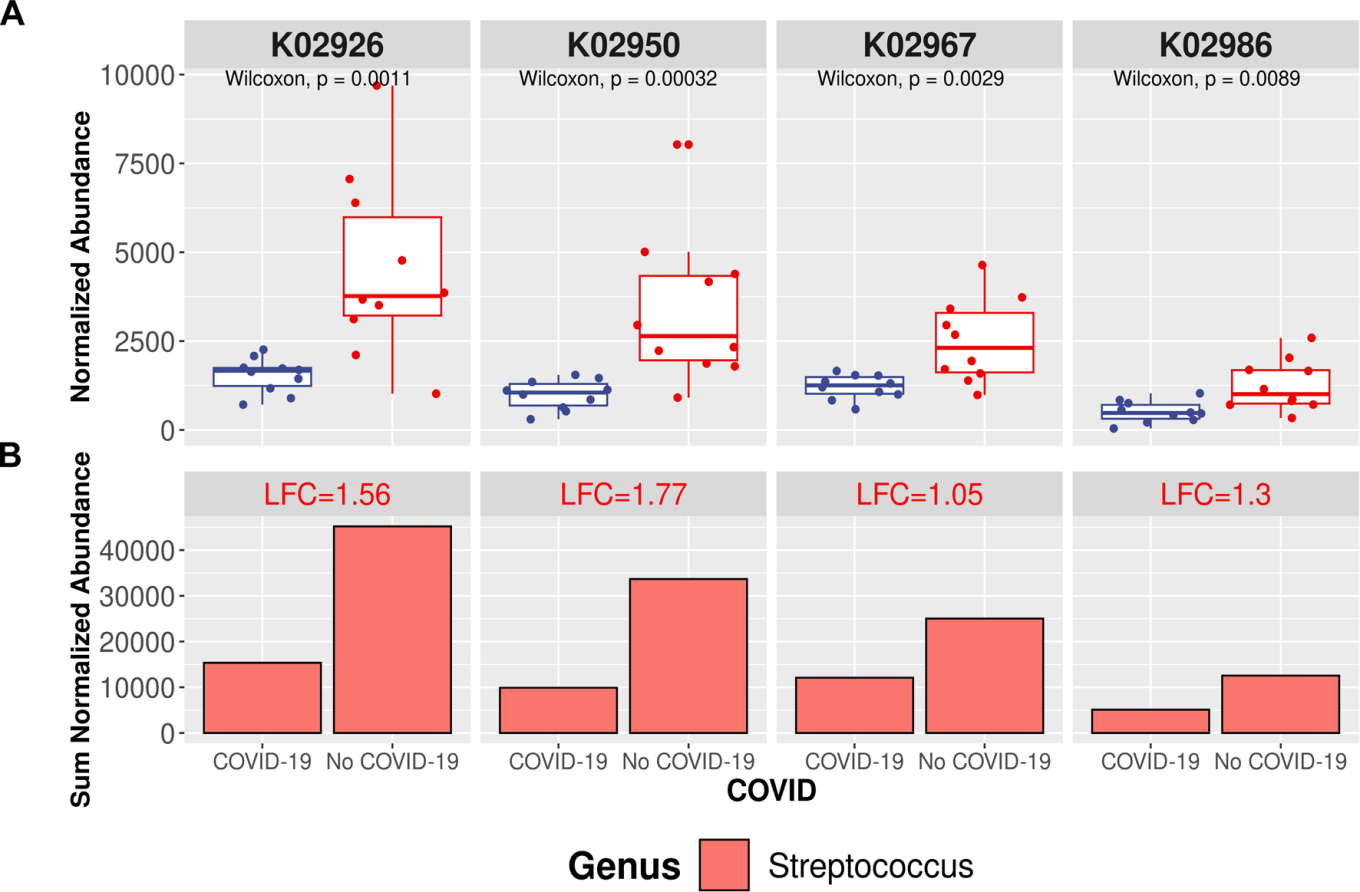
Comparison of DE KOs in saliva of patients affected by COVID-19 and not affected in terms of Wilcoxon test (upper panel) and bacteria-contributing *genera* (lower panel). Box plots show the differentially presented KOs according to a p-value < 0.01. Bar plots represent the sum of the normalized abundance of the proteins associated with them (**A**) and the taxonomy at *the genus* level. **B** LFC (log2-fold change (FC)) means log_2_(A/B), where A is the sum of the normalized abundance of the proteins contributing to the KO for patients affected by COVID-19 and not affected in the case of B

Because different microbiota features have been described as relevant actors for disease outcomes, we decided to explore some of these traits to understand the link between the active salivary microbiome and COVID-19 disease. According to the strict confidence intervals detailed in the materials and methods section, we identified and quantified a total of 500 microbial proteins (Additional file 1: Table S4B). We compared, using the ANOSIM test, the abundance of individual microbial proteins, as well as their KO and taxonomy, in the saliva microbiome according to the presence of SARS-CoV-2 infection: COVID-19^+^ (Mcov & Scov) and COVID-19^˗^ (NcNs & NcSus). While no statistically significant difference was found when examining protein expression profiles, we identified 4 translation-related Kyoto Encyclopedia of Genes and Genomes (KEGG) Orthology (KO) genes overexpressed in the COVID-19^˗^ group: K02926, K02950, K02967, and K02986 (Fig. 4, p-value ≤ 0.001). Their expression was decreased in the COVID-19^+^ group, suggesting that the translational machinery, and thus microbial protein biosynthesis, of the microbiota in COVID-19^+^ patients might be impaired. This result agrees with the fact that protein synthesis has been found to be altered in nasopharyngeal swabs from COVID-19 patients [11]. Notably, proteins associated with KOs overexpressed in the COVID-19^˗^ group were assigned to bacteria from the *Streptococcus* genus. This suggests that although this taxonomic group has been found to be highly abundant in patients with COVID-19 [21], at the functional level, its involvement in pathogenesis should be limited.

### Targeted correlations of protein expression with ACE2 activity in plasma

**Fig. 5:**
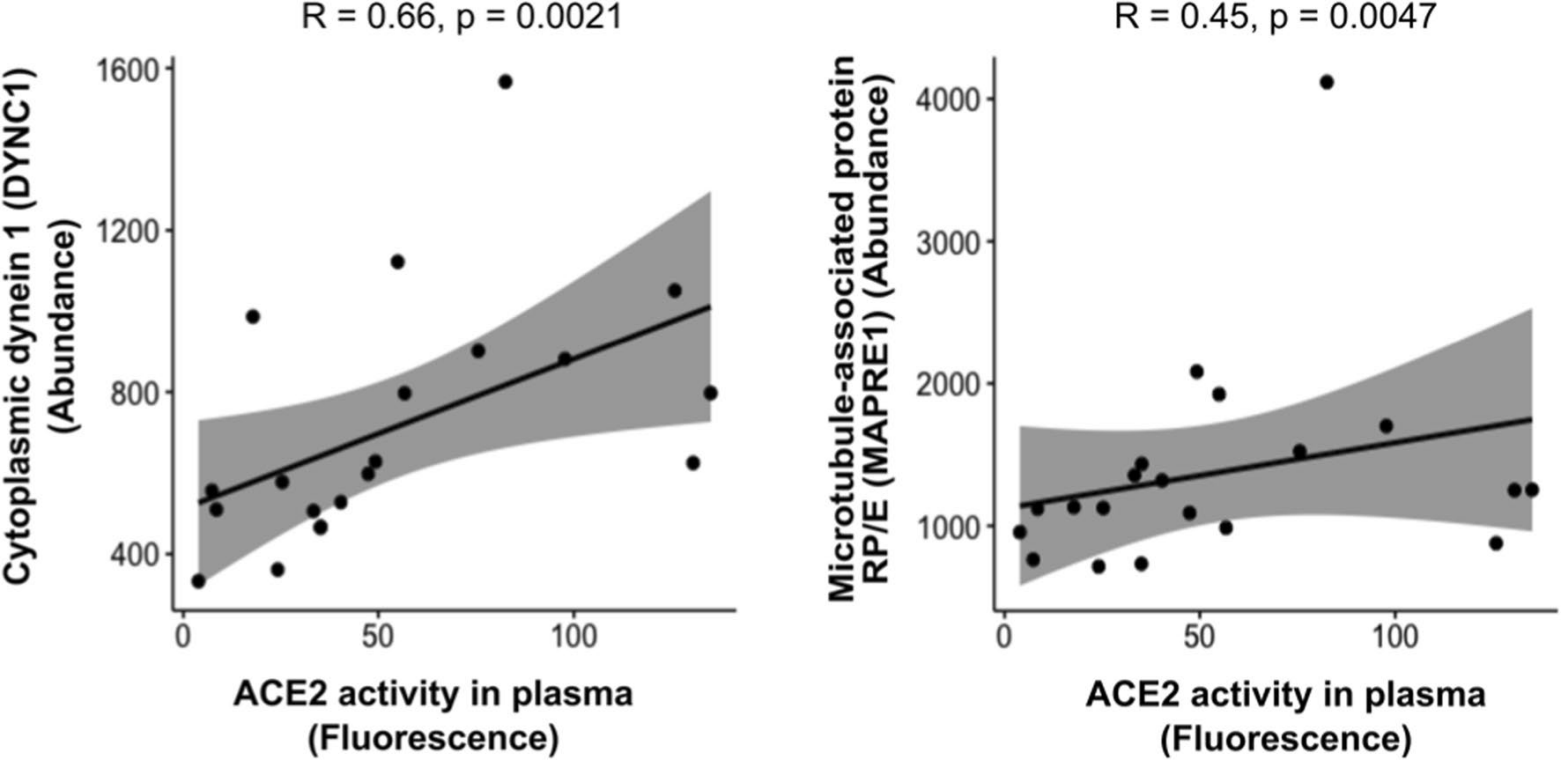
Significant correlations of factors identified in Metascape and IPA analysis with previous ACE2 fluorescence levels quantified in plasma. Graphs of correlations for Dynein 1 (DYNC1) and microtubule-associated protein RP/E (MAPRE1) abundances (normalized) with ACE2 fluorescence are shown. As described in Material and Methods, abundances were calculated based on TMT label intensities normalized using an Internal Standard control (Additional file 1: Table S1) and fluorescence was measured at 420 nm (arbitrary units). Linear trends were analyzed, and their fit is depicted by the Pearson R coefficient and the p-values in each graph

Finally, because angiotensin-converting enzyme 2 (ACE2) is the molecular target for SARS-CoV-2 cell entry, we analyzed the correlations between the DE proteins consistently identified in the Metascape analysis and matching with the IPA analysis (FAM20A, MAOB, DYNC1, MAPRE1, and DNAH17) with ACE2 activity. In Fig. 5, we show the positive and significant correlations found between the DYNC1 and MAPRE1 proteins and ACE2 activity. These two proteins are related to protein transport through microtubules in the cytoplasm of cells, possibly affecting viral replication by allowing viral proteins to reach the Golgi apparatus or endoplasmic reticulum to replicate and translate their proteins. Furthermore, both proteins were decreasingly expressed in the Non-COVID and nonsusceptible groups (Additional file 1: Table S1), indicating a potential role of DYNC1 and MAPRE1 in SARS-CoV-2 susceptibility by affecting ACE2 levels in plasma.

## Discussion

Patients with COVID-19 have been reported to have significant alterations in oral (tongue-coating) and gut microbial diversity [21–23], as well as alterations in the expression level of proteins from fecal microbiomes [24, 25], plasma human proteomes [26], nasopharyngeal swabs [27–29] and semen samples [30]. Proteome profiling of nasopharyngeal swabs from COVID-19 patients further revealed alterations in proteins implicated in the innate immune response, viral assembly, and exocytosis [31]. To the best of our knowledge, only one study has focused on the application of untargeted mass spectrometry to compare the saliva proteome of SARS-CoV-2-infected and non-COVID-19 individuals. Although differences in the expression of a human protein related to the defense/immunity protein, an immunoglobulin, a translational protein, and a protease inhibitor were observed, no clear link with the mechanisms of pathogenesis and response to SARS-CoV-2 could be established [32].

This exploratory study aimed to describe DE proteins that could be related to COVID-19 disease susceptibility. Several proteomic analyses have been published to date to find causal candidates or drug targets to defeat SARS-CoV-2 infection, but very few have been performed in saliva, the only sample from the main infection sites of the virus. Furthermore, our study includes an extraordinary group of study, the nonsusceptible group, formed by a small group of heavily exposed healthcare workers who were not infected by SARS-CoV-2. This is important to understand possible specific mechanisms that might be responsible for some protective status of certain people who do not get infected although they are heavily exposed to the virus.

We are aware of the main limitations of our study: the sample size was small due to the difficulty at that time in selecting patients for each specific condition; it is limited to the first viral variant and cannot be extrapolated to other variants; and there might be confounding factors such as diabetes, age and sex. Diabetes has been shown to affect the salivary proteome in previous studies [33–36] and 5 subjects in our study had this condition. Age is also an important factor, since the proteome of adults over 60 years is different from younger individuals [37–39] and we had age differences in the moderate COVID-19 group. However, we did not see these differences in the main comparison of groups: NcNs vs NcSus. Furthermore, as a quality control of our study, to determine the identified human proteins with the highest confidence we filtered and selected only the proteins appearing in two experiments with two internal standards, and even doing that, we obtained similar or even higher numbers compared to previous studies in nasopharyngeal swabs [11] and in saliva samples [32]). It also constitutes one of the best studies reporting the combined human proteome and microbial meta-proteome in saliva samples, since no previous study reported alterations in canonical human and microbial pathway analysis in saliva samples. However, other technical validations, such as western blot, were not performed, since they are out of the scope of this particular study and samples were limited.

**Fig. 6:**
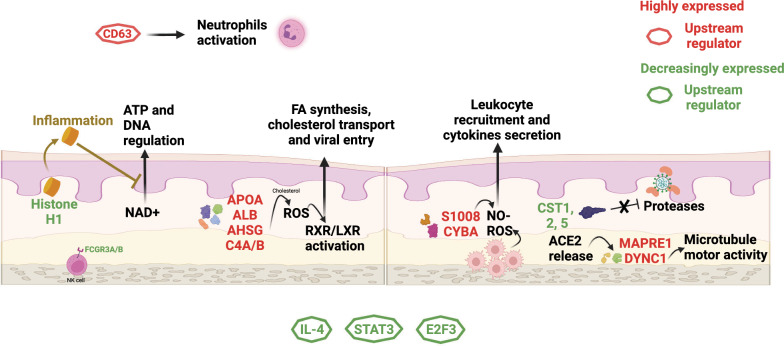
Summary of the main pathways and DE proteins in nonsusceptible individuals identified in this study. Highly expressed proteins are depicted in red, and decreasingly expressed proteins are depicted in green. An octagon shape has been added to the proteins that have roles as upstream regulators of processes involved in SARS-CoV-2 pathogenesis and that came up in our analysis. (This figure can go to Additional file 1)

When we analyzed the DE proteins of the nonsusceptible group compared to the susceptible group, we found some interesting findings. The most important observations are summarized in Fig. 6 for clarification and will be described in the following paragraphs.

When analyzing the described groups, the NcNs and NcSus comparison yielded the most significant differences (Fig. 1). Focusing on this comparison, we found that the main canonical pathway was the LXR/RXR activation pathway. The liver X receptor forms a heterodimer with the retinoid X receptor, and they participate in the regulation of lipid metabolism, inflammation, and cholesterol transportation [40, 41]. Furthermore, its inhibition has been associated with prolonged viral RNA shedding [42]. Related to this pathway, we found molecules mainly synthesized in the liver and related to HDL synthesis and cholesterol transport, immune modulators, or regulators of viral entry, such as APOA1, AHSG, or C4A. Another important canonical pathway in our analysis is the NO production pathway. The production of nitric oxide and ROS by activated macrophages is essential to the control of infections and is regulated by IFN-gamma. Specifically, this pathway has been related to the pathophysiology of the cytokine storm and severe COVID-19 [43, 44]. Some DE proteins related to these functions were increasingly expressed in the nonsusceptible group, such as S100A8 and CYBA. S100 proteins are part of the calprotectin dimer, which has been related to COVID-19 severity [45, 46], and CYBA has been described as an interactor of the Nef protein of HIV-1 [47]. The dimer SA8/A9 is released by neutrophils and macrophages during inflammation, so its overexpression before infection could have a protective effect [48]. Another significant canonical pathway was the NAD signaling pathway. NAD is the active metabolite of vitamin B3 and is essential for cellular processes of energy metabolism, cell protection, and biosynthesis [49]. Moreover, its function was previously related to inflammation and coagulation present in COVID-19 [50, 51]. Decreased expression of histone H1 appears to be related to this pathway in our analysis. This could be due to a protective role, since histone H1 acts as a sensor of NAD^+^, which is related to ATP production and DNA regulation. It has also been shown that extracellular histone H1 is released in response to inflammatory challenges, reducing NAD^+^ levels and thus maintaining low NAD^+^ protection roles [52, 53]. Furthermore, in our previous study with these same samples, we found a link with energy metabolism and biosynthesis, where some compounds, such as citrulline, citric acid, and histidine decrease, and BAIBA, phenylalanine, and 2-AB increase were related to mitochondrial and liver dysfunction as a consequence of hypoxemia associated with SARS-CoV-2 severity [54], and some of the DE proteins found in this study are also involved in metabolic pathways, such as cholesterol-NO-ROS, cholesterol-LXR or tryptophan-NAD^+^ pathways.

The main upstream regulators identified in our analysis and associated with organismal death were IL-4, STAT3, and E2F3, whose activity was predicted to be decreased by IPA analysis based on the expression of the proteins in our dataset. IL-4 is related to IFN-gamma, IL-17A, and STAT6, all factors involved in T-cell differentiation to helper T cell and vitamin D3 receptor [55, 56]. STAT3 is a factor related to IL-6 and NF-KB, is known as an antiviral and proinflammatory mediator [57], and has been largely associated with COVID-19 disease severity [57–63]. E2F3 is a transcription factor of genes involved in the cell cycle and is known to be regulated by some viruses, such as HIV, HCV, and EBV [64–66]. Moreover, E2F3 is usually downregulated by miRNAs to avoid cell proliferation, which is usually very related to viral infections [64, 67, 68]. Previous analysis of nasopharyngeal swabs from COVID-19 patients found altered expression of proteins involved in the innate immune response, cell death and inflammation in COVID-19-infected patients. Among these proteins, only IFN and STAT proteins were also found to be associated with COVID-19 in saliva samples, which suggests different responses in oral (saliva) and nasopharyngeal environments during SARS-CoV-2 infection [11].

Regarding specific proteins, in nonsusceptible individuals, we observed higher expression in general of the S100 proteins (calprotectin) family, which are calcium- and zinc-binding proteins produced by immune cells and involved in several cellular processes, including cycle progression and differentiation, immune regulation, inflammation, etc. [48, 69, 70]. Increased expression of S100A6 is related to signal transduction, ion transmembrane transport, positive regulation of fibroblast proliferation, or protein binding [71]. Specifically, elevated levels of proteins of the calprotectin family have been related to COVID-19 severity [44, 45]. Decreased expression of the histone H1 family is related to NAD signaling, protein kinase A signaling, and sirtuin signaling pathways [72], which are all related to cellular metabolism and growth. The histone H1 family acts as a regulator of transcription through chromatin remodeling, nucleosome spacing, and DNA methylation, which are associated with the NAD signaling pathway. Decreased expression of FCGR3A/B, which are IgG receptors of NK cells and polymorphonuclear neutrophils, is implicated in antibody-dependent enhancement of viral infections. Highly expressed TRIM56, a previously described antiviral host that confers resistance to human coronavirus OC43, dengue virus, and yellow fever virus (15). Mucin and AQP5 are related to the calpain protease family, which has been used as a target against SARS-CoV-2 infection by M protease inhibitors (29).

Another relevant group of proteins found to be associated with NcNs is the cystatin family, including cystatin-SN (CST1), cystatin-SA (CST2), CST3, and cystatin-D (CST5). All these proteins were found to be significantly downregulated in the NcNs and COVID + groups when compared with NcSus, except for CST3, which was only downregulated in the NcNs group. Cystatin 5 (or D) is an active cysteine protease inhibitor that plays a protective role against specific proteases present in the oral cavity [73]. It has also been previously described as overexpressed during other virus infections, such as hepatitis B infection [74]. High concentrations of some cystatins have been associated with higher COVID-19 severity and mortality [75]. Thus, the decrease in the levels of cystatins in nonsusceptible individuals may be a protective mechanism that helps to maintain the peptidase activity level, which could help to fight potential viral infections.

Another interesting protein that appeared as highlighted in the networks related to acute respiratory disorder and syndrome in our analysis for the nonsusceptible group was the CD63 protein. CD63 + activated neutrophils have been related to the acute phase of SARS-CoV-2 infection [76] and to ZIKV infection protection as a marker in EVs [77].

Finally, a correlation analysis with ACE2 levels in plasma showed a significant correlation with DYNC1 and MAPRE1. DYNC1 acts as a motor for the intracellular retrograde motility of vesicles and organelles along microtubules through ATPase activity. This activity has been related to RNA binding and microtubule motor activity and is associated with neuropathies [78]. Regarding SARS-CoV-2, the use of motor proteins, kinesins, and dynein, prominent in neuronal pathways, has been shown to be essential for viral migration through olfactory nerves to the brain [79]. The olfactory lobe, a part of the brain that translates smell responses, is the starting point of dysfunction, which partly explains the early signs of inability to smell, i.e., anosmia [80, 81]. Dynein is part of the endosome unit that mediates viral entry and then localizes and releases it in the right place of the cell, facilitating viral replication [82]. MAPRE1 regulates microtubule polymerization and mediates cargo transport. This role has been shown during HIV infection [83]. The fact that the expression of these proteins is decreased in the nonsusceptible group in our analysis and that they correlate positively with plasma ACE2 activity, which has been described before as increased following SARS-CoV-2 infection [84], supports that they might play a specific role during SARS-CoV-2 infection.

In summary, this study is the first to explore, through the analysis of DE proteins, possible alterations in canonical human and microbial pathways in saliva samples and their association with SARS-CoV-2 infection [64–66]. Although several studies from plasma, semen or cell proteomes have been published, the biological processes found to be altered in saliva differ from those in other types of samples, and thus, this study provides a complementary view into COVID-19 pathogenesis. Furthermore, since microbiota proteins are being revealed as potentially relevant factors in the outcome of some diseases [85–87], our exploration of a potential role in COVID-19 pathogenesis might seed some interesting facts.

## Materials and methods

### Study design and setting

Participants confirmed SARS-CoV-2 (COVID-19^+^ group) infection by polymerase chain reaction (PCR) from nasopharyngeal swabs, sputum, or lower respiratory tract secretions within the first 7 days from the onset of symptoms. Then, they were classified according to clinical severity as follows: moderate disease, defined as the presence of bilateral radiologic infiltrates or opacities and clinical assessment requiring supplemental oxygen; and severe disease, defined as the development of acute respiratory distress syndrome [31]. Participants without SARS-CoV-2 (non-COVID group) were asymptomatic subjects with a negative PCR from nasopharyngeal swabs. These individuals were considered “susceptible” to COVID-19 since they had positive IgG for SARS-CoV-2 or previous COVID-19 confirmed by PCR from nasopharyngeal exudate (non-COVID susceptible, NcSus). In contrast, the group considered as nonsusceptible adults were healthy healthcare workers who were on duty for at least 3 months in COVID-19 wards or intensive care units and reported at least three high-risk exposures to SARS-CoV-2 [88] without having experienced symptoms suggestive of SARS-CoV-2 infection, were persistently negative for SARS-CoV-2 PCR testing and did not have SARS-CoV-2 IgM and IgG in plasma (non-COVID nonsusceptible, NcNs). Additionally, this nonsusceptible group differs from the Non-COVID group in that they did not get infected either at any time in the next 5 months, in contrast to the Non-COVID group, in which the 5 individuals eventually became infected at some point during the next 5 months. The most frequent exposure was largely unprotected exposure to aerosol-generating procedures or patient secretions and close contact without facemasks with other confirmed cases of COVID-19. We measured SARS-CoV-2 antibodies by indirect chemiluminescence immunoassay (Vircell, Granada, Spain). Samples were coded for further proteomic analysis as indicated in the “Coding” sheet in Additional file 1: Table S1.

### Sample collection and treatment

Unstimulated saliva samples (5 ml aprox) were collected consistently across all participant, from fasting patients early in the morning, to ensure minimal variation in the saliva composition. Any additional support or device were used to avoid contaminations with other secretions, such as sputum.

Cryopreserved (− 80 ℃) saliva samples were inactivated, reduced and alkylated with a 1:1 (v:v) volume of 2X denaturing buffer (10% sodium dodecyl sulfate (SDS) + 200 mM triethylammonium bicarbonate (TEAB) + 10 mM tris(2-carboxyethyl)phosphine (TCEP) + 10 mM chloroacetamide (CAA)), followed by incubation at 60 ℃ for 30 min. The total amount of protein was quantified by PIERCE 660 nm reagent (compatible with ionic detergents). Protein digestion on S-Trap columns (Protifi) was performed following the manufacturer’s instructions with minor changes [89]: 50 µg of each protein sample was digested at 37 ℃ overnight using trypsin protease (ratio 1:15, enzyme-protein). A pool of all digested samples was included as an internal standard (IS) in each experiment. After overnight incubation, the peptides were eluted, dried in a speed-vac and quantified by fluorimetry (QuBit). For TMT-11plex labeling, the dried digests were distributed into two labeling experiments: 20 µg of each of the 11 samples per experiment were labeled with the corresponding TMT 11-plex tag. After two hours, the labeled digests from each batch were pooled and fractionated.

### Basic-pH fractionation using SDB-RPS STAGE Tips

The fractionation of TMT-labeled peptides was performed using in-house-made STAGE tips prepared from SDB-RPS solid-phase extraction disks (Empore^™^) similar to previously reported protocols [90, 91]. The STAGE tip was prepared according to the protocol previously described by our research group [92]. Briefly, 12 pieces were cut off from SDB-RPS solid-phase extraction disks with the assistance of a 16-gauge blunt end needle and packed into a 200 µL tip. The STAGE tip was inserted onto the top of a 2-mL tube using an in-house-made adapter and activated with 100 µL MeOH and centrifuged at 900 × g for 3 min. The tip was then conditioned with steps of 100 µL 50% acetonitrile (ACN) and 0.1% formic acid (FA) and centrifuged, followed by three equilibration steps with 0.1% FA. After that, TMT-labeled peptides (80 µg) were reconstituted in 100 µL 1% FA (pH < 3) and loaded onto the STAGE tip. The sample was centrifuged at 900 × g for 5 min, and the collected flow-through was loaded again to improve the peptide recovery yield. The STAGE tip was washed with 100 µL 0.1% FA, followed by 100 µL H_2_O. The elution was carried out using a 10-stepwise elution with 100 µL of 5 mM ammonium formate buffer and increasing acetonitrile concentrations (0, 5.0%, 7.5%, 10.0%, 12.5%, 15.0%, 17.5%, 20%, 25%, and 60%). Fractions were dried in a speed vacuum and frozen until further processing.

### Analysis by liquid chromatography coupled to mass spectrometry OE-240

Each fraction (10 fractions in total) of the TMT-labeled experiments was quantified by fluorimetry (QuBit), and a 1 µg aliquot of each fraction was subjected to 1D-nano LC‒ESI‒MS/MS analysis using an Ultimate 3000 nano HPLC system (Thermo Fisher Scientific) coupled online to an Orbitrap Exploris^™^ 240 mass spectrometer (Thermo Fisher Scientific). Tryptic peptides (200 ng/µL) were eluted onto a 50 cm × 75 μm Easy‐spray PepMap C18 analytical column at 45 ℃ and separated at a flow rate of 300 nL/min using a 90 min gradient ranging from 2 to 95% mobile phase B (mobile phase A: 0.1% FA; mobile phase B: 80% ACN in 0.1% FA). The loading solvent was 2% ACN in 0.1% FA. In any case, we ruled out any carry-over effect since between the fractions of each experiment, two 40 min blanks were injected and systematically checked.

Data acquisition was performed using a data-dependent top-20 method in full scan positive mode, scanning 375 to 1200 m/z. Survey scans were acquired at a resolution of 60,000 at m/z 200, with a normalized automatic gain control (AGC) target (%) of 300 and a maximum injection time (IT) in AUTO. The top 20 most intense ions from each MS1 scan were selected and fragmented via higher-energy collisional dissociation (HCD). The resolution for HCD spectra was set to 45,000 at m/z 200, with an AGC target of 100 and a maximum ion injection time in AUTO. Isolation of precursors was performed with a window of 0.7 m/z, exclusion duration (s) of 45 and HCD collision energy of 30. Precursor ions with single, unassigned, or six and higher charge states from fragmentation selection were excluded.

Data obtained by mass spectrometry were analyzed with Proteome Discoverer (v2.4.0.305) using four search engines (Mascot (v2.7.0), MsAmanda (v2.4.0), MsFragger (v3.1.1) and Sequest HT) and a target/decoy database built on one side from sequences in the *Homo sapiens* and *SARS-CoV-2* proteomes at UniProt Knowledgebase (20210225, 20,464 sequences, respectively) and on the other side, the high‐quality reference human gut microbiome database (87), formerly called Integrated Genome Catalog, which contains approximately 10 million human microbiome protein sequences with functional and taxonomic annotations. All searches were configured with dynamic modifications for TMT reagents (+ 229.163 Da) on lysine and N-termini of the peptide, pyrrolidone from Q (− 17.027 Da) and oxidation of methionine residues (+ 15.9949 Da), and static modification as carbamidomethyl (+ 57.021 Da) on cysteine, monoisotopic masses, and trypsin cleavage (max 2 missed cleavages). The peptide precursor mass tolerance was 10 ppm, and the MS/MS tolerance was 0.02 Da. The false discovery rate (FDR) cutoff for proteins, peptides, and peptide spectral match (PSM) peptides was kept at 1%.

Quantitation was also performed in Proteome Discoverer using the “Reporter Ions Quantifier” feature in the quantification workflow using the following parameters: unique + razor peptides were used for quantitation, the coisolation threshold was set at 50%, the signal-to-noise ratio of reporter ions was 10, and normalization and scaling were performed considering the total peptide amount and the control (IS) average, respectively. Protein groups (master proteins) with an FDR lower than 1% and with abundance values in both IS were considered for quantitation. No imputation method was used because only proteins quantified in all channels and present in both internal standards (IS) were included. The protein ratio was calculated considering the protein abundance, and the hypothesis test was based on a t test (background based). Then, an adjusted p-value ≤ 0.05 of the abundance ratio using Benjamini‒Hochberg was set to determine the DE proteins (up- and downregulated) in paired comparisons (Additional file 1: Table S1D–I), Volcano plots and PCA (performed in Proteome Discover software).

To further understand the specific roles related to SARS-CoV-2 infection of the DE human proteins identified in our analysis, we used the following tools: Qiagen’s IPA software [93] and the Metascape website [12]. Ingenuity Pathway Analysis (IPA) software from Qiagen uses a proprietary algorithm to generate causal networks from input data. The algorithm takes into account various factors, including published literature, pathway databases, and experimental evidence, to identify causal relationships between molecules in the input dataset. It estimates whether proteins in a given network are increased or decreased based on the direction of change in the expression levels of their corresponding genes. Metascape compares user-introduced gene or protein lists with all analyses from –omic technologies related to SARS-CoV-2 samples collected from different databases. For this study, we uploaded our DE protein list from the comparison of non-COVID-susceptible vs non-COVID-susceptible groups to the Metascape website and selected the best enrichment from RNA-seq and proteomics analysis.

Preprocessing of the protein intensities for the selected microbial proteomes was performed as follows: (a) the protein abundance was calculated according to the Peptide Spectrum Matches (PSMs), which has been commonly used as a relative quantitation score of the proteins in a complex mixture based on protein coverage by the peptide matches in a database search result; (b) the identified proteins were assigned to KO pathways (87–89), and the relative abundance of each KO was obtained by the sum of the relative abundances of all proteins assigned to each of the identified KO [94]; (c) the differential abundance of proteins and KO among groups were obtained using Wilcoxon test p-values, using R programming language [95]. We considered DE KOs between groups those with a p-value ≤ 0.01.

### ACE-2 activity measurement

Plasma samples were collected in EDTA tubes and cryopreserved at − 80 ℃. After thawing, ACE2 activity was measured in batches through a fluorometric assay using a synthetic ACE2-specific substrate (Mca-APK(Dnp); Reactomix S.L., Granada, Spain) that is metabolized to a fluorescent compound in the presence of a functional enzyme. Samples were incubated with the buffer solution (150 nM Tris–HCl pH 7.5, 200 nM NaCl, 10 µM ZnCl2, protease inhibitor) and the substrate (portions 10-99-1) at room temperature for 16 h. We quantified each sample's fluorescence using Varioskan lux (Thermo), a fluorescence reader with an excitation of 320 nm and an emission of 420 nm. Measurements in duplicate showed a coefficient of variation of 5.1%. The protein concentration, measured colorimetrically by the Pierce^™^ 660 nm Protein Assay reagent (Thermo Scientific, USA) for all samples, was on average 1.58 ± 0.07 µg protein/µL, with no significant differences across samples. Therefore, the differences in ACE2 activity have biological significance and are not due to a bias in the amount of protein in each sample. According to this technique, increased fluorescence indicates increased ACE2 activity.

We used R software to calculate Spearman’s coefficients for the targeted correlation analyses for ACE2 plasma activity and the six proteins consistently identified in the METASCAPE analysis (library corrplot).

## Significance of the study

Although several proteomics analyses have been performed in plasma samples from COVID-19 patients to understand the role of host factors in disease progression, information about the role of the human salivary proteome is scarce. Furthermore, although several studies have addressed the differences between different degrees of the disease, little is known about those people who are less susceptible to SARS-CoV-2 infection even having been heavily exposed to the virus. In this study, we analyzed a unique cohort of unvaccinated people to address these two specific questions. The salivary proteome of nonsusceptible to COVID-19 patients compared to that of susceptible patients or infected patients showing different degrees of the disease (moderate and severe) was obtained. Then we compared our data with previously published datasets related to COVID-19 and analyzed bacterial-produced proteins, which have been shown to have regulatory roles in different diseases, regarding The Microbiome Database. Finally, we studied correlations with the plasma activity of the molecular target of SARS-CoV-2, ACE2, and found a link with two proteins related to protein transport through microtubules in the cytoplasm (DYNC1 and MAPRE1). Our study identifies new pathways involved in SARS-CoV-2 susceptibility and pathogenesis that will be useful to design strategies to combat the COVID-19 pandemic.

### Supplementary Information

**Additional file 1: Figure S1.** Main interaction network from IPA analysis for DE proteins in saliva of the comparison of Moderate COVID (Mcov) vs Non-COVID nonsusceptible (NcNs). **Figure S2.** Main interaction networks from IPA analysis for DE proteins in saliva of the comparison of Moderate COVID (Mcov) vs Non-COVID susceptible (NcSus). **Figure S3.** Main interaction network from IPA analysis for DE proteins in saliva of the comparison of severe COVID (Scov) vs non-COVID susceptible (NcSus). **Figure S4.** Main interaction networks from IPA analysis for DE proteins in saliva of the comparison of severe COVID (Scov) vs non-COVID nonsusceptible (NcNs). **Figure S5.** Main interaction networks from IPA analysis for DE proteins in saliva of the comparison of severe COVID (Scov) vs moderate COVID (Mcov). **Figure S6.** Comparisons with other databases were performed to infer the main transcription factors, transcription factor targets, cell types, and tissues. **Table S1.** Quality-filtered nonredundant human proteins identified from saliva samples, their expression level, and their functional annotation. **Table S2.** Lists of DE human proteins identified from saliva samples that were used for functional analysis based on IPA. **Table S3.** Discriminatory pathways associated with DE human proteins herein identified in saliva samples of all conditions compared to those in all the -omic studies published related to COVID-19 by using Metascape. **Table S4.** Quality-filtered nonredundant microbial-produced proteins identified from the saliva samples and their expression levels.

## Data Availability

The datasets supporting the conclusions of this article are included within the article (and its additional files). The mass spectrometry proteomics data have been deposited to the ProteomeXchange Consortium via the PRIDE partner repository [96] with the dataset identifiers PXD032333 and10.6019/PXD032333.

## Abbreviations

ACN: Acetonitrile
ACE2: Angiotensin-converting enzyme 2
AGC: Automatic gain control
CAA: Chloroacetamide
DE: Differentially expressed
FA: Formic acid
FDR: False discovery rate
GO: Gene Ontology
HCD: Higher-energy collisional dissociation
IPA: Ingenuity Pathway Analysis
IS: Internal standard
KEGG: Kyoto Encyclopedia of Genes and Genomes
Kos: KEGG Ontologies
LC‒ESI‒MS/MS: Liquid Chromatography Electrospray Ionization Tandem Mass Spectrometric
Mcov: Moderate COVID-19
NcNs: Non-COVID and nonsusceptible individuals
NcSus: Non-COVID and susceptible individuals
PSMs: Peptide spectral matches
ROS: Reactive oxygen species
Scov: Severe COVID-19
SDB-RPS: Reversed-phase Stage-Tips
TCEP: Tris(2–24 carboxyethyl) phosphine
TEAB: Triethylammonium bicarbonate
TMT: Tandem mass tags
