# Supplementary material for: Proteomic snapshot of saliva samples predicts new pathways implicated in SARS-CoV-2 pathogenesis
Source: Clin Proteomics. 2024 May 22;21:37. doi: 10.1186/s12014-024-09482-9 (PMC11112864; doi:10.1186/s12014-024-09482-9)
Supplement: Supplementary file 1 — Additional file 1: Figure S1. Main interaction network from IPA analysis for DE proteins in saliva of the comparison of Moderate COVID (Mcov) vs Non-COVID nonsusceptible (NcNs). Figure S2. Main interaction networks from IPA analysis for DE proteins in saliva of the comparison of Moderate COVID (Mcov) vs Non-COVID susceptible (NcSus). Figure S3. Main interaction network from IPA analysis for DE proteins in saliva of the comparison of severe COVID (Scov) vs non-COVID susceptible (NcSus). Figure S4. Main interaction networks from IPA analysis for DE proteins in saliva of the comparison of severe COVID (Scov) vs non-COVID nonsusceptible (NcNs). Figure S5. Main interaction networks from IPA analysis for DE proteins in saliva of the comparison of severe COVID (Scov) vs moderate COVID (Mcov). Figure S6. Comparisons with other databases were performed to infer the main transcription factors, transcription factor targets, cell types, and tissues. Table S1. Quality-filtered nonredundant human proteins identified from saliva samples, their expression level, and their functional annotation. Table S2. Lists of DE human proteins identified from saliva samples that were used for functional analysis based on IPA. Table S3. Discriminatory pathways associated with DE human proteins herein identified in saliva samples of all conditions compared to those in all the -omic studies published related to COVID-19 by using Metascape. Table S4. Quality-filtered nonredundant microbial-produced proteins identified from the saliva samples and their expression levels. [file 12014_2024_9482_MOESM1_ESM.zip › Supp Info Feb24/Sup Inf_Proteomic SARS-CoV2.docx]

# Supporting Information for Proteomic snapshot of saliva samples predicts new pathways implicated in SARS-CoV-2 pathogenesis

**Elena Moreno^1,2†^, Sergio Ciordia^3†^, Fátima Millhano Santos^3 †^, Daniel Jiménez^1^, Javier Martínez-Sanz^1,2^, Pilar Vizcarra^1,2^, Raquel Ron^1,2^, Matilde Sánchez-Conde^1,2^, Rafael Bargiela^4^, Sergio Sanchez-Carrillo^5ϒ^, Santiago Moreno^1,2,6^, Fernando Corrales^3§^, Manuel Ferrer^5§^, Sergio Serrano-Villar^1,2§^**

^1^Department of Infectious Diseases, Hospital Universitario Ramón y Cajal, IRYCIS, 28034 Madrid, Spain.

^2^CIBERINFEC, Instituto de Salud Carlos III, 28029, Madrid, Spain

^3^Functional Proteomics Laboratory, Centro Nacional de Biotecnología (CNB), CSIC, 28049 Madrid, Spain.

^4^Centre for Environmental Biotechnology, School of Natural Sciences, Bangor University, LL57 2UW Bangor, United Kingdom

^5^Instituto de Catalisis y Petroleoquimica (ICP), CSIC, 28049 Madrid, Spain.

^6^Facultad de Medicina, Universidad de Alcalá de Henares, 28801 Alcalá de Henares, Madrid, Spain

**†These authors have contributed equally to this work and share the first authorship**

**^ϒ^Current address, Centro de Biologia Molecular Severo Ochoa (CBM), CSIC-UAM, 28049 Madrid, Spain**

**^§^These authors share senior authorship**

**Correspondence:**Elena Moreno, PhD. Department of Infectious Diseases, Hospital Universitario Ramon y Cajal (IRYCIS). Carretera de Colmenar Viejo, Km 9.100, 28034 Madrid, Spain. E-mail: emolmo@salud.madrid.org

**Content:**

Figure S1. Main interaction network from IPA analysis for DE proteins in saliva of the comparison Moderate COVID (Mcov) vs Non-COVID non-susceptible (NcNs).

Figure S2. Main interaction networks from IPA analysis for DE proteins in saliva of the comparison Moderate COVID (Mcov) vs Non-COVID susceptible (NcSus).

Figure S3. Main interaction network from IPA analysis for DE proteins in saliva of the comparison Severe COVID (Scov) vs Non-COVID susceptible (NcSus).

Figure S4. Main interaction networks from IPA analysis for DE proteins in saliva of the comparison Severe COVID (Scov) vs Non-COVID non-susceptible (NcNs).

Figure S5. Main interaction networks from IPA analysis for DE proteins in saliva of the comparison Severe COVID (Scov) vs Moderate COVID (Mcov).

Figure S6. Comparisons with other databases to infer the main transcription factors, transcription factor targets, cell types, and tissues.

Table S1. Quality-filtered non-redundant human proteins identified from saliva samples, their expression level, and their functional annotation.

Table S2. Lists of DE human proteins identified from saliva samples that were used for functional analysis based on the IPA analysis.

Table S3. Discriminatory pathways associated to DE human proteins herein identified in saliva samples of all conditions, compared to those in all the -omic studies published related to COVID-19 by using Metascape.

Table S4. Quality-filtered non-redundant microbial-produced proteins identified from the saliva samples and their expression level.


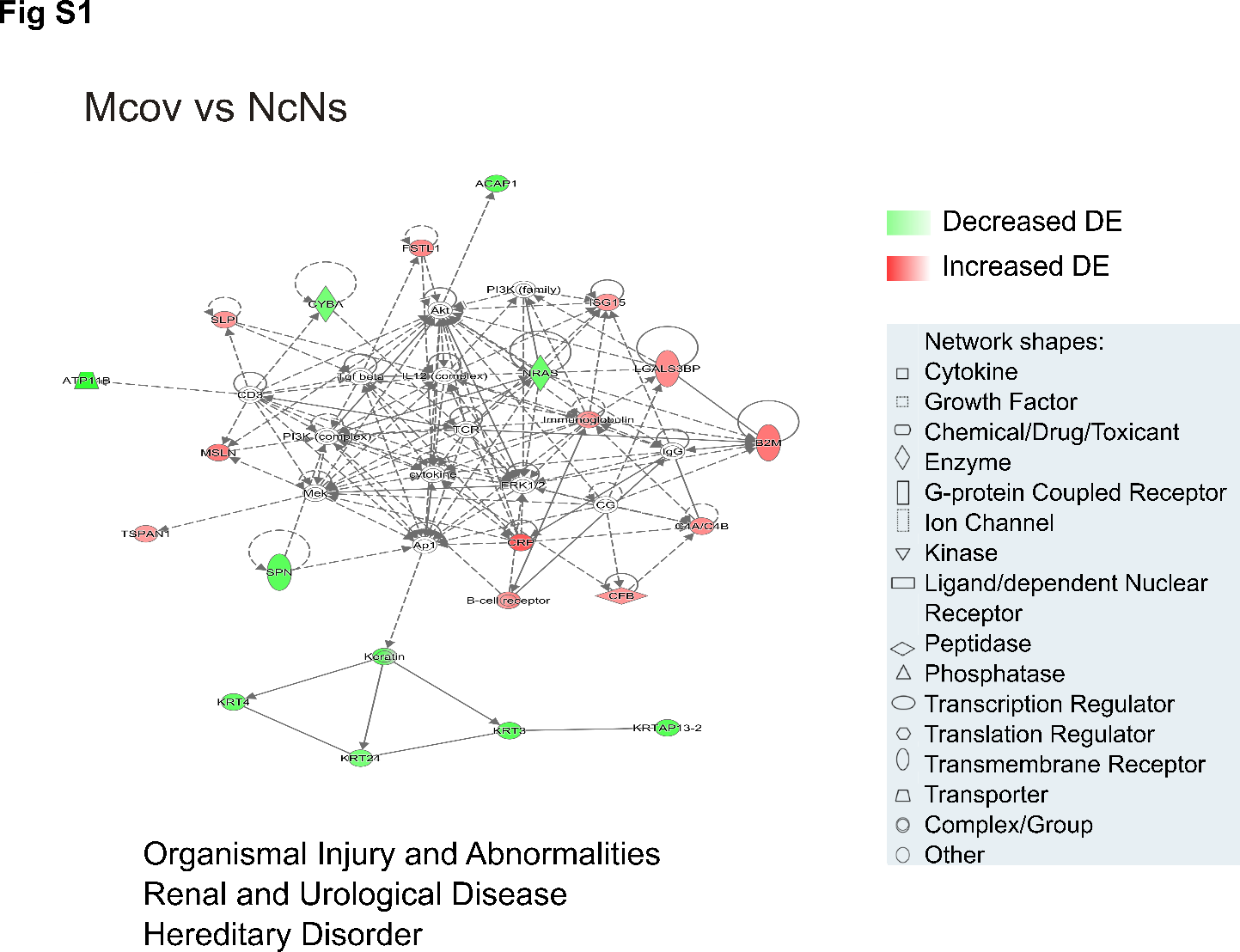


**Figure S1. Main interaction network from IPA analysis for DE proteins in saliva of the comparison Moderate COVID (Mcov) vs Non-COVID non-susceptible (NcNs).** Symbols depict different types of molecules indicated in the blue box and red and green gradients represent an increased differential expression or decreased differential expression respectively. Arrows connecting symbols depict connections between signaling events with transcription effects.


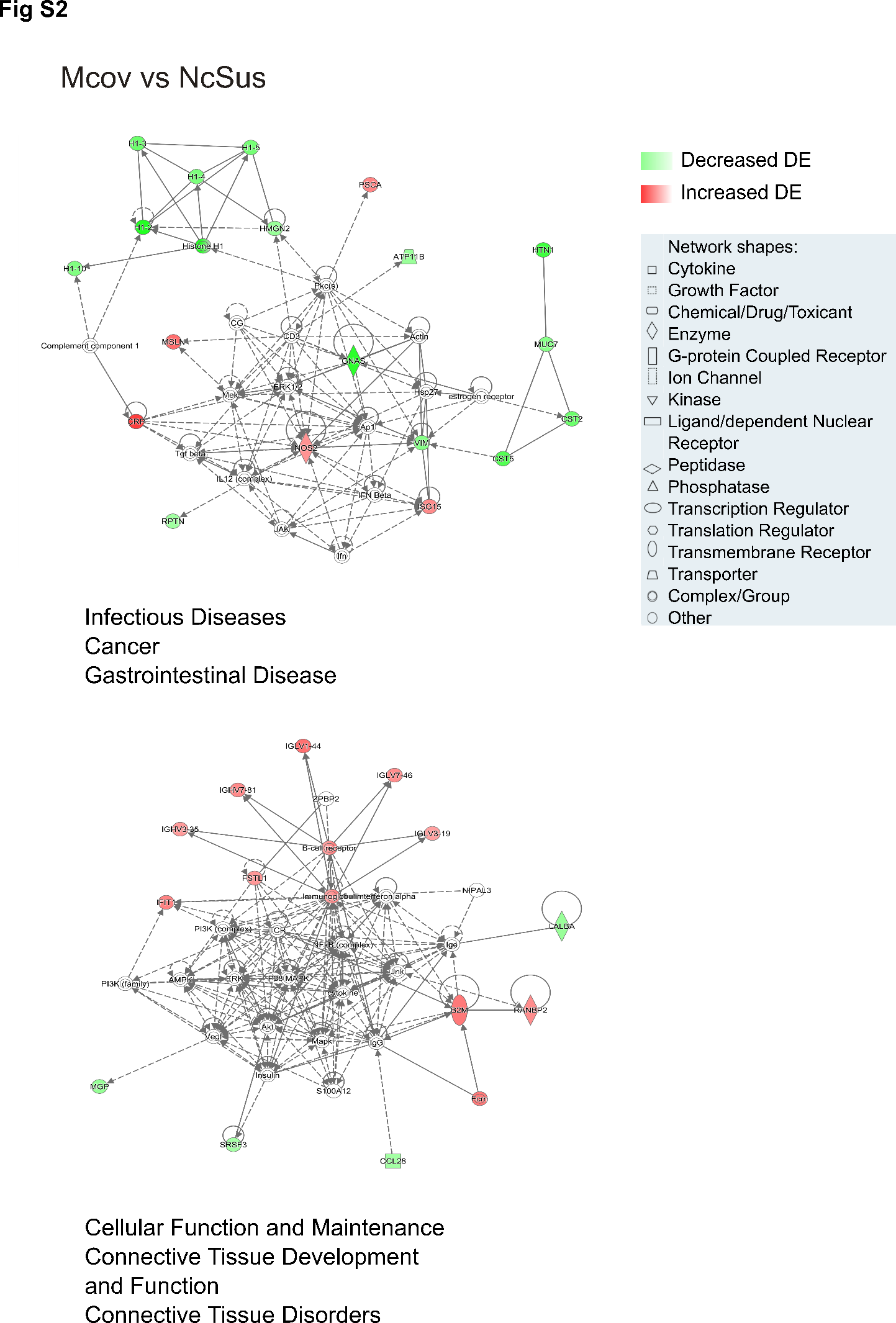


**Figure S2. Main interaction networks from IPA analysis for DE proteins in saliva of the comparison Moderate COVID (Mcov) vs Non-COVID susceptible (NcSus).** Symbols depict different types of molecules indicated in the blue box and red and green gradients represent an increased differential expression or decreased differential expression respectively. Arrows connecting symbols depict connections between signaling events with transcription effects.


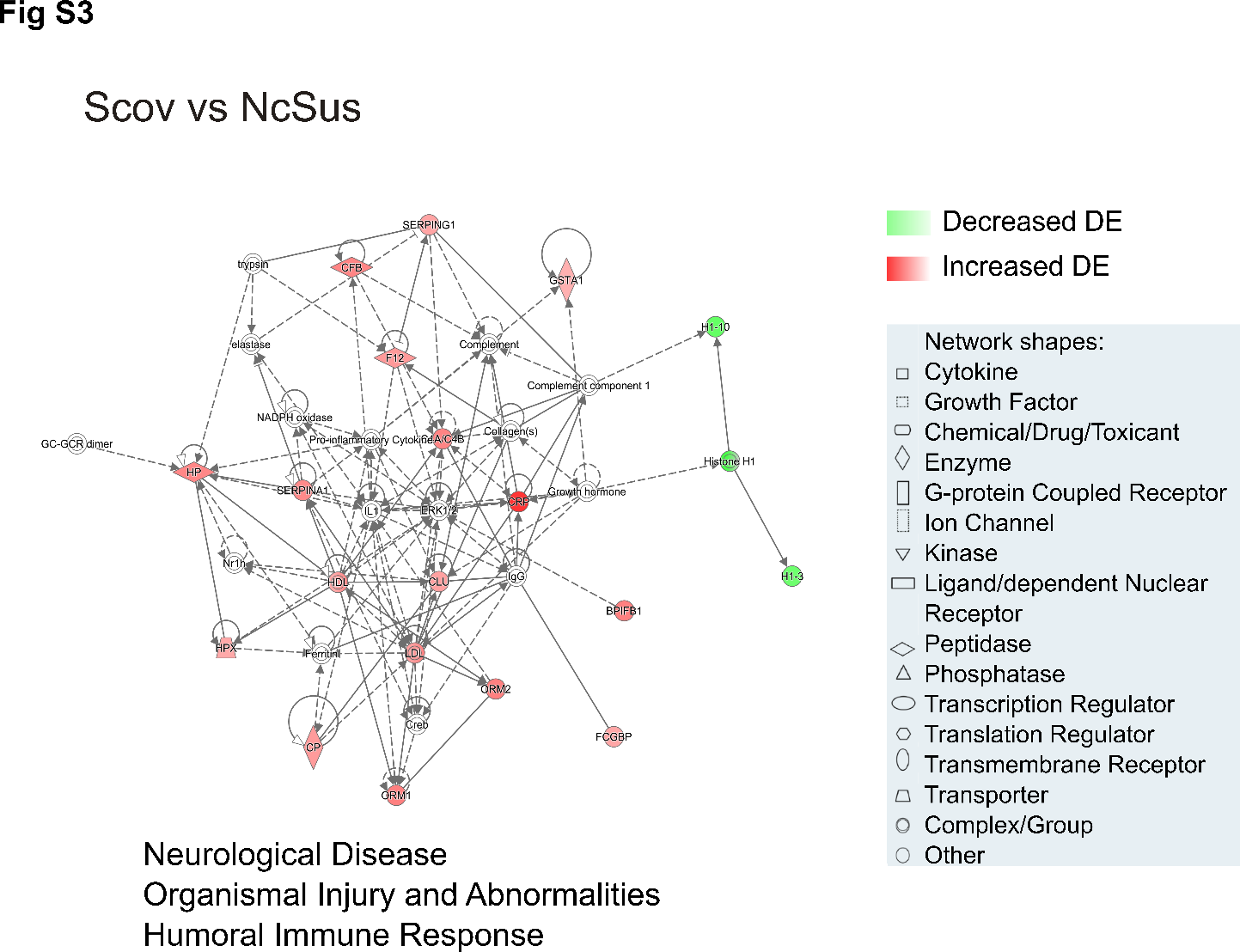


**Figure S3. Main interaction network from IPA analysis for DE proteins in saliva of the comparison Severe COVID (Scov) vs Non-COVID susceptible (NcSus).** Symbols depict different types of molecules indicated in the blue box and red and green gradients represent an increased differential expression or decreased differential expression respectively. Arrows connecting symbols depict connections between signaling events with transcription effects.

**
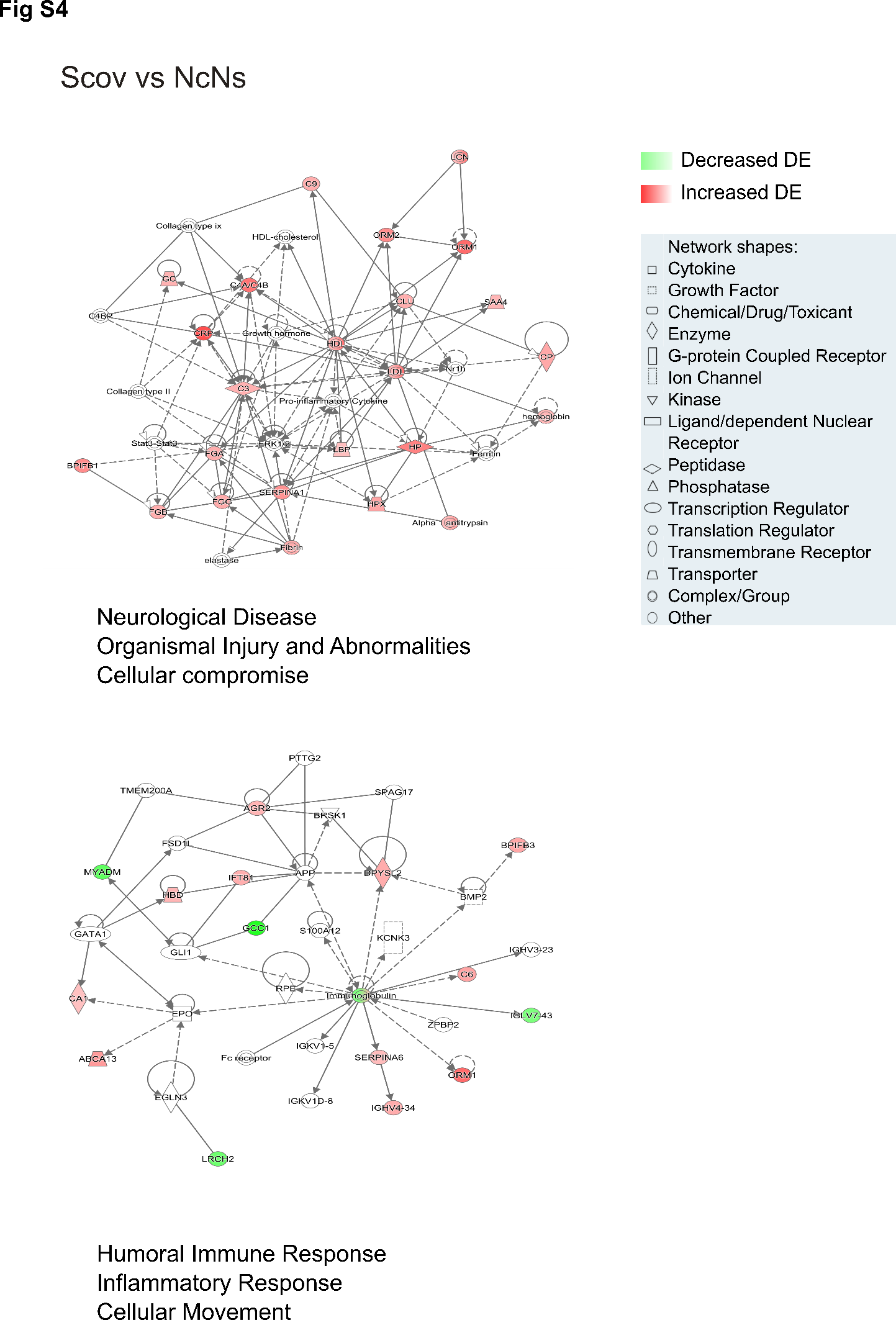
**

**Figure S4. Main interaction networks from IPA analysis for DE proteins in saliva of the comparison Severe COVID (Scov) vs Non-COVID non-susceptible (NcNs).** Symbols depict different types of molecules indicated in the blue box and red and green gradients represent an increased differential expression or decreased differential expression respectively. Arrows connecting symbols depict connections between signaling events with transcription effects.


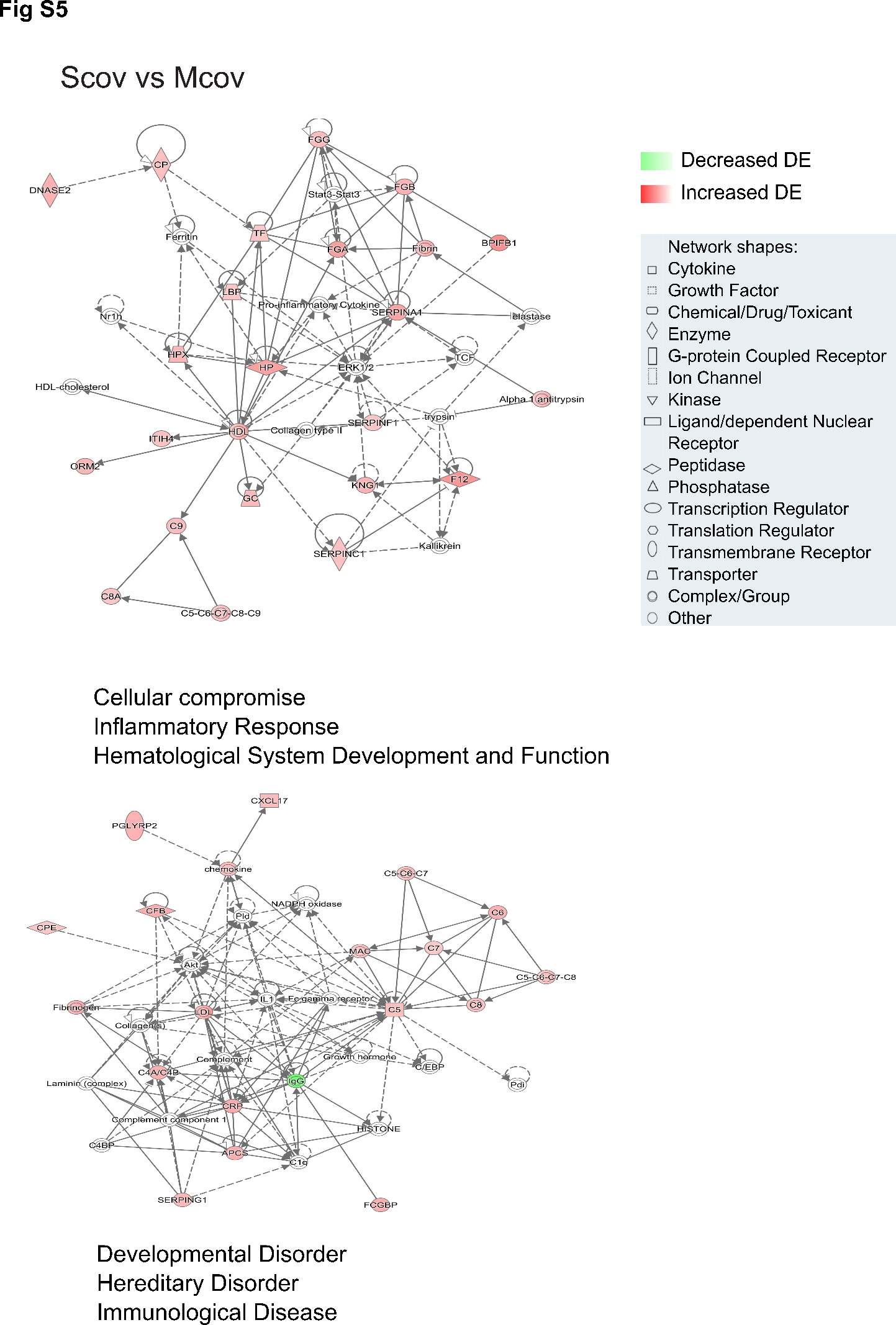


**Figure S5. Main interaction networks from IPA analysis for DE proteins in saliva of the comparison Severe COVID (Scov) vs Moderate COVID (Mcov).** Symbols depict different types of molecules indicated in the blue box and red and green gradients represent an increased differential expression or decreased differential expression respectively. Arrows connecting symbols depict connections between signaling events with transcription effects.


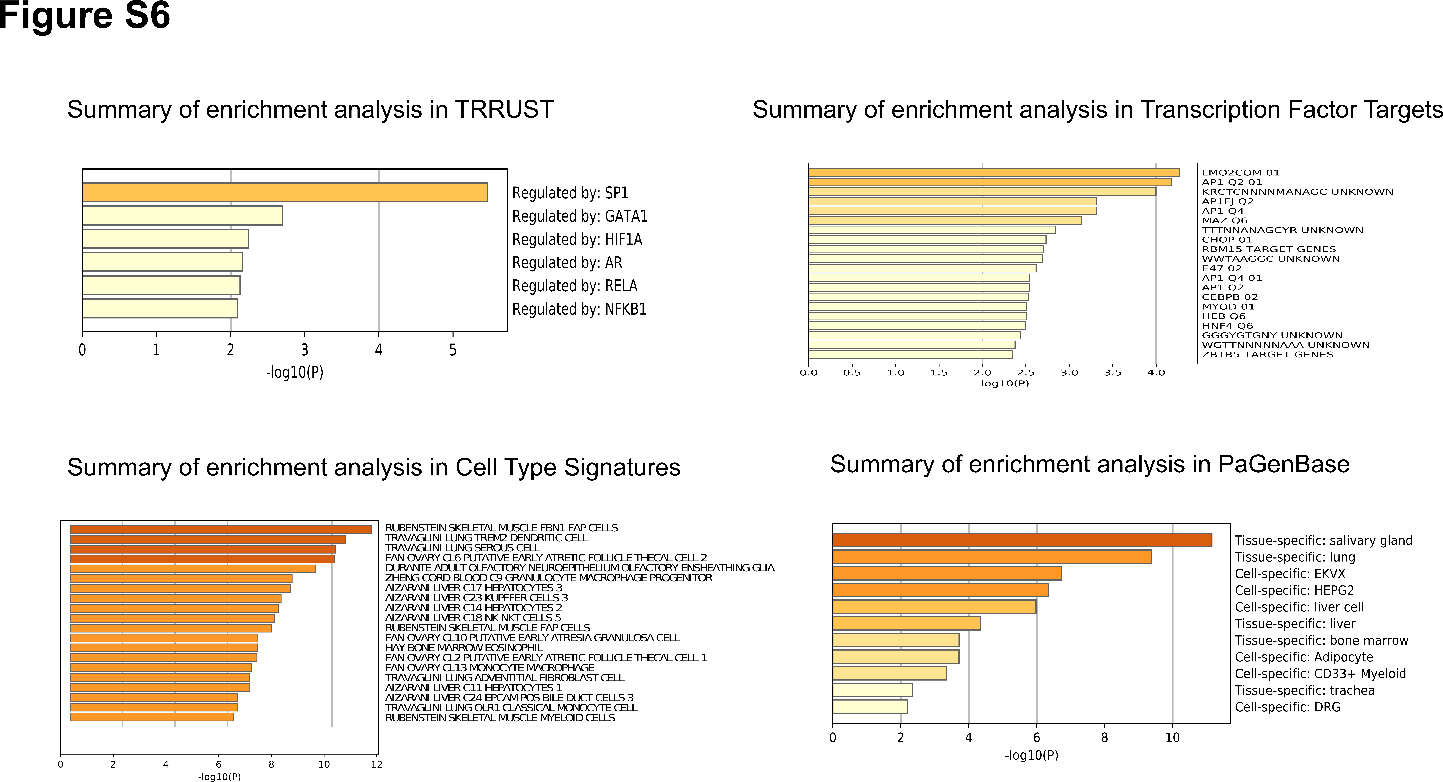


**Figure S6. Comparisons with other databases were performed to infer the main transcription factors, transcription factor targets, cell types, and tissues.** Results of the main terms obtained in the analysis performed with Metascape of our list of proteins compared to other specific databases.

**Table S1. Quality-filtered human proteins identified from saliva samples, their expression level, and their functional annotation.** The following information is provided in a number of sub-tables:

**Description:** this table briefly summarizes the number of proteins unambiguously identified and quantified by high-throughput proteomic profiling in all saliva samples, and those found to be DE between the different study groups. In all cases, the strict criteria used for the identification, quantification and DE analysis, are highlighted.

**A, Coding:** includes the internal IDs for each experiment, the TMT reagents used for labeling (TAG), sample and sample code and the groups to which each associated.

**B, Protein-List_ID:** includes the list of the proteins identified using a FDR cutoff for proteins, peptides, and peptide spectral matches (PSMs) peptides at 1%. For each protein, the following information is provided: the ID and annotation according to UniProt database, the molecular weight (MW), the isoelectric point (pI), the experimental q-value (HIGH confidence indicates q-value <0.01), the sum PEP score that corresponds to the score calculated based on the posterior error probability (PEP) values of the peptide spectrum matches (PSM), the score for each of the search engines used, the number of PSMs, the number of peptides assigned to each protein, and the protein sequence coverage by identified peptides. By a color code, the presence (green squares) or absence (red squares) of each protein in each sample is indicated.

**C, Protein-List_QUANT-OnlyIS:** includes the list of proteins quantified in both IS and TMT experiments. For each protein, the following information is provided: the ID and annotation according to UniProt database, the molecular weight (MW), the isoelectric point (pI), the experimental q-value (HIGH confidence indicates q-value <0.01), the sum PEP score that corresponds to sum of the normalized abundances of the peptides assigned to the same protein (unique + shared peptides), the score for each of the search engines used, the number of fragmented spectra (PSMs), the number of peptides assigned to each protein, and the protein sequence coverage by identified peptides. By a color code, the presence (green squares) or absence (red squares) of each protein in each sample is indicated. The abundance of each protein is calculated as the sum of the normalized abundances of the peptides assigned to the same protein (unique + shared peptides), based on which the abundance ratio and q-value are calculated for each comparison. Differential expression is color-coded: Proteins up-regulated with q-value<0.01 are represented at dark red and down-regulated at dark green.

**D-I, DEPs:** lists the DE proteins between the different study groups. Comparisons as follows: D. DEPs_Mcov-NcNs, E. DEPs_Scov-NcNs, F. DEPs_Scov-Mcov, G. DEPs_NcNs-NcSus, H. DEPs_Mcov-NcSus, I. DEPs_Scov-NcSus. The information provided in each comparison is as detailed in sub-table C.

Because its extensive size this table is provided as separate Excel table.

**Table S2. Results of the functional analysis based on the IPA analysis of the DE human proteins found in saliva samples.** The comparisons between the different groups are located in different sheets in the file. Each analysis shows the identified canonical pathways, predicted upstream regulators, causal networks, diseases and biological functions, toxic functions, regulator effects, predicted networks, toxicity list and analysis-ready molecules for the DE proteins of each comparison. Rows highlighted in yellow match with the categories depicted in Figure 2. For further information on each of these categories, see Qiagen website [1]. The top 10 hits selected for Figure 2 have been highlighted in yellow and have been selected based on the highest –log_10_(p-value) with cutoff >0.000 together with a z-score value >0. Details about networks depicted in Figure 2 are given in this table for each comparison, going to the different sections of the table highlighted in dark green: Canonical Pathways, Upstream Regulators, Causal Networks, Diseases and Bio Functions, etc.

Because its extensive size this table is provided as separate Excel table.

**Table S3. Discriminatory pathways associated to DE human proteins herein identified in saliva samples of all conditions, compared to those in all the -omic studies published related to COVID-19 by using Metascape.** The table summarizes the results of the metascape analysis for the comparison of the list of the hits obtained comparing the groups NcNs vs NcSus with all previously published data of viral protein interactions in Metascape, selected for having the most relevant matches with the list of protein herein identified. User-provided gene identifiers are first converted into their corresponding H. sapiens Entrez gene IDs using the latest version of the database (last updated on 2022-01-01). If multiple identifiers correspond to the same Entrez gene ID, they will be considered as a single Entrez gene ID in downstream analyses. Each gene list is assigned a unique color, which is used throughout the analysis. The following information is provided in different sheets:

- - - 1. Annotation: The following gene annotations are provided:
         1. Gene Symbol: Primary HUGO gene symbol.
         2. Description: Short description.
         3. Biological Process (GO): Descriptions are summarized based on the gene ontology database, where up to three most informative GO terms are kept.
         4. Kinase Class (UniProt): Detailed kinase classes.
         5. Protein Function (Protein Atlas): Protein Function (Protein Atlas)
         6. Subcellular Location (Protein Atlas): Subcellular Location (Protein Atlas)
         7. Drug (DrugBank): Drug information for the given gene as a target.
         8. Canonical Pathways: Ontology, Canonical Pathways
         9. Hallmark Gene Sets: Ontology, Hallmark Gene Sets
      2. Enrichment: enrichment analysis applying the standard accumulative hypergeometric statistical test to identify ontology terms, where input genes show significant presence. Including ontology terms from Broad's MSigDB, as well as automatically cluster resultant terms to reduce redundancy. Enriched GO terms then ordered by the relevance of their function according to the LogP value (p<0.01). Description of the functions and the main genes associated with them are provided.
      3. MCODE: 3 MCODE components (Molecular Complex Detection algorithm) are displayed separately from the previous Enrichment analysis of the 121 proteins list. The algorithm identifies densely connected network neighborhoods, where such neighborhood component is more likely to be associated with a particular complex or functional unit than the rest of the network, characterizing its biological significance. The color code matches with the colors in Figure 3B.

Because its extensive size this table is provided as separate Excel table.

**Table S4. Quality-filtered microbial-produced proteins identified from the saliva samples and their expression level.** The information provided in the different sub-tables is as detailed in Table S1. Proteomic information and annotation for quality-filtered non-redundant proteins include information about gene length, gene status, cohort origin, taxonomic affiliation (phylum and/or genus level), frequency and individual frequency, KO, eggNOG, KO category and KO title are indicated. Unknown phylum refers to proteins assigned to bacteria but from which no clear phylogenetic assignation could be obtain (either because its novelty or the small size of the DNA fragment containing the protein). NA, means “Phylogeny Not Assigned” and refers to proteins with no clear phylogenetic assignation. In all cases, functional annotations and protein information were assigned as detailed elsewhere [2].

Because its extensive size this table is provided as separate Excel table.

**References**

[1] Qiagen, Qiagen IPA analysis features, Website. (2022). https://digitalinsights.qiagen.com/products-overview/discovery-insights-portfolio/analysis-and-visualization/qiagen-ipa/features/.

[2] S. Ruiz‐Ruiz, S. Sanchez‐Carrillo, S. Ciordia, M.C. Mena, C. Méndez‐García, D. Rojo, R. Bargiela, E. Zubeldia‐Varela, M. Martínez‐Martínez, C. Barbas, M. Ferrer, A. Moya, S. Ruiz-Ruiz, S. Sanchez-Carrillo, S. Ciordia, M.C. Mena, C. Méndez-García, D. Rojo, R. Bargiela, E. Zubeldia-Varela, M. Martínez-Martínez, C. Barbas, M. Ferrer, A. Moya, Functional microbiome deficits associated with ageing: Chronological age threshold, Aging Cell. 19 (2020) e13063. https://doi.org/10.1111/acel.13063.
